# Serum hypoxia-inducible factor-1α and uterine artery Doppler ultrasound during the first trimester for prediction of preeclampsia

**DOI:** 10.1038/s41598-021-86073-w

**Published:** 2021-03-23

**Authors:** Wasinee Tianthong, Vorapong Phupong

**Affiliations:** Placental Related Diseases Research Unit, Department of Obstetrics and Gynecology, Faculty of Medicine, Chulalongkorn University, Rama IV Road, Pathumwan, Bangkok, 10330 Thailand

**Keywords:** Biomarkers, Diseases

## Abstract

The objective of this study was to determine the predictive value of serum hypoxia-inducible factor-1α (HIF-1α) combined with uterine artery Doppler in singleton pregnancy during 11–13^+6^ weeks of gestation for preeclampsia. This prospective observational study was conducted in singleton pregnant women at 11–13^+6^ weeks of gestation who visited the King Chulalongkorn Memorial Hospital, Faculty of Medicine, Chulalongkorn University for antenatal care between February 2019 and May 2020. Serum HIF-1α levels and uterine artery Doppler ultrasound were performed. Pregnancy outcomes were recorded. The sensitivity, specificity, positive predictive value (PPV), and negative predictive value (NPV) of these tests at the optimal cut-off values were determined to predict preeclampsia. A total of 385 participants were analyzed. Of these, 31 cases had preeclampsia (8.1%), and 6 cases of them had early-onset preeclampsia (1.6%). Preeclamptic women had significantly higher serum HIF-1α levels than normal pregnant women (median 1315.2 pg/ml vs. 699.5 pg/ml, p < 0.001). There was no difference in the mean pulsatility (PI) of the uterine artery. Serum HIF-1α levels were higher than 1.45 multiple of median for the gestational age as a cut-off value for predicting preeclampsia; the sensitivity, specificity, PPV, and NPV were 66.7%, 71.5%, 17.2%, and 96.2%, respectively. When a combination of abnormal serum HIF-1α levels and abnormal uterine artery Doppler PI (above the 95th percentile) were used as a predictive value to predict preeclampsia, the sensitivity, specificity, PPV, and NPV were 74.2%, 67.2%, 16.6%, and 96.8%, respectively. This study showed that the serum HIF-1α levels with or without uterine artery Doppler at 11–13^+6^ weeks of gestation were effective in predicting preeclampsia.

## Introduction

Hypertensive disease of pregnancy is considered one of the most frequent obstetric complications that affects up to 10% of pregnant women^1^. Preeclampsia, a serious type of pregnancy-induced hypertension, is a member of the deadly obstetrical triad along with infection and hemorrhage as well as a leading cause of maternal and neonatal morbidities^2,3^. The global incidence of preeclampsia is 2–8%. Furthermore, there are estimated 50,000–60,000 women worldwide who die of preeclampsia each year^1^.

The etiology of preeclampsia remains unknown, but it is thought to be the consequence of impaired trophoblastic invasion of the maternal spiral arteries due to an imbalance between levels of angiogenic factors and hypoxia-induced oxidative stress^4,5^. According to previous studies, a defective placental invasion results in uteroplacental insufficiency and is associated with adverse pregnancy outcomes such as fetal growth restriction and preeclampsia, especially during the early and severe form of this condition^4,5^.

The American College of Obstetricians and Gynecologists (ACOG) and the National Institute for Care and Health Excellence (NICE) recommend identifying patients who are at high risk of developing preeclampsia based on their medical history. Pregnant women who are at high risk of developing preeclampsia are prescribed low-dose aspirin to minimize the incidence and severity of the disease^6–8^. Although this traditional approach using clinical risk factors has limited predictive ability and can identify only 30% of pregnant women who develop preeclampsia, it is the only currently recommended screening method^6,9,10^. Nowadays, researches are focused on finding new markers that have high predictive value to provide early intervention in order to reduce morbidity and mortality. Many screening tests, such as those using biochemical markers and ultrasound markers, were investigated whether it can predict preeclampsia or not, but none of them have been found to be predictably reliable, valid, and suitable for routine clinical use^10^.

Hypoxia-inducible factor-1 (HIF-1) is a heterodimer consisting of two subunits, α and β. It is a key transcription factor that mediates cellular response to low oxygen tension. HIF-1α levels are rapidly reduced under normal oxygen condition. But they are increased in hypoxic environments^11,12^. Placentation develops in a low-oxygen environment during early pregnancy before 10 weeks of gestation when there is limited blood flow into the intervillous space. HIF-1α is upregulated to maintain trophoblasts in a proliferative, noninvasive, and immature phenotype. After that, intervillous blood flow is increased at 10–12 weeks of gestation leading to normoxic conditions and lower levels of HIF-1α. The differentiation of extravillous trophoblasts occurs and they physiologically invade the myometrial segment of the uterus. For pregnancies complicated by preeclampsia, HIF-1α expression remains abnormally elevated and the trophoblasts development remains arrested at an immature stage, causing a shallow trophoblast invasion^11,13–18^. Previous studies found that pregnant women with preeclampsia had high blood levels of HIF-1α compared to normal healthy controls^19,20^. Galbiati et al. found that HIF-1α was significantly higher in women who later developed preeclampsia compared to women who did not^21^.

Serum HIF-1α levels have never been used to predict preeclampsia. Therefore, this study assessed the predictive value of serum HIF-1α combined with uterine artery Doppler in singleton pregnancy during 11–13^+6^ weeks of gestation for preeclampsia and other pregnancy complications, such as fetal growth restriction, preterm delivery, and perinatal death.

## Materials and methods

This prospective observational study was performed at the Department of Obstetrics and Gynecology, King Chulalongkorn Memorial Hospital, Faculty of Medicine, Chulalongkorn University, Bangkok, Thailand, between February 2019 and May 2020. This study was approved by the Research Ethics Committee of the Faculty of Medicine, Chulalongkorn University. All procedures were performed in accordance with the relevant guidelines and regulations of the Institutional Review Board. This study has been performed in accordance with the Declaration of Helsinki. All subjects gave written informed consent.

Singleton pregnant women at a gestational age of 11–13^+6^ weeks, aged between 20 and 45 years who came to the antenatal clinic were invited to participate in this study. Gestational age was determined by the last menstrual period and confirmed by the measurement of the fetal crown-rump length at the first-trimester ultrasound. Women who used aspirin as a prophylaxis for preeclampsia were excluded from the study. In addition, pregnant women who were diagnosed to have fetal structural or chromosomal abnormalities were excluded.

Since there is a lack of data on the predictive value of HIF-1α for the prediction of preeclampsia, the sample size was calculated based upon the hypothesis that the sensitivity of serum HIF-1α combined with uterine artery PI in preeclampsia prediction was 80% with 20% allowable error. This indicated that the study needed 15 cases of preeclampsia. The incidence of preeclampsia at our institute was 4.9%. After we adjusted the calculation using our institute’s incidence of preeclampsia and a loss to follow-up rate of 20%, a minimum of 368 women were required for this study.

Written informed consent was obtained from all eligible participants. The questionnaire on maternal age, medical history, parity, and obstetric history was completed by interviewing each participant. Maternal weight and height were measured to calculate the body mass index (BMI).

Blood pressure was measured by validated automated devices (Microlife AG, 9443 Widnau, Switzerland) after at least 5 min of rest in the seated position. The mean arterial pressure was recorded.

Uterine artery Doppler was assessed transabdominally by a single sonographer using ultrasonographic machines (GE Voluson E10, GE Medical Systems, Zipf, Austria) with a convex probe AB 2–7 MHz. Initially, a mid-sagittal section of the uterus and cervix was obtained, and the probe was then tilted sideways to identify the uterine artery blood flow along both sides. Two-millimeter gate pulse-wave Doppler was positioned on the branch of the uterine artery close to the internal cervix, with an insonation angle < 30°. At least three identical waveforms with the peak systolic velocity more than 60 cm/s were obtained. The pulsatility index (PI) was recorded for each side^22^. An abnormal Doppler of the uterine artery result was defined as having a mean PI more than the 95th percentile for each gestational age^22^.

Venous blood samples (10 ml) were drawn from each participant into a non-heparinized tube. Serum was separated by centrifugation at 2500 rounds per minute for 10 min and frozen at − 80 °C until assay. After recruitment was completed, HIF-1α concentration was measured using the HIF 1-α enzyme-linked immunosorbent assay (ELISA) (Cloud-Clone Corp, Massachusetts, TX, USA)^23^.

SPSS software version 22.0 (IBM, New York, USA) was used for statistical analysis. Results were presented as mean with standard deviation, median with interquartile range (IQR), sensitivity, specificity, positive predictive value (PPV), negative predictive value (NPV), and relative risk, with a 95% confidence interval. The optimal cut-off values for HIF-1α levels were calculated using the receiver operator characteristic curve. A Chi-square test, Fisher’s exact test, unpaired *t* test, and Mann–Whitney *U* test were used when appropriate. A p value < 0.05 was considered to be statistically significant.

## Results

Four hundred and seven pregnant women were enrolled into this study. Twenty-two women were excluded due to spontaneous miscarriage prior to 20 weeks (7 cases), lost to follow-up (13 cases), or fetal anomalies were diagnosed during the second trimester (2 cases). The data from a total of 385 women were analyzed. Thirty-one participants were diagnosed with preeclampsia (8.1%), and 6 of them had early-onset preeclampsia (1.6%).

The demographic data and pregnancy outcomes among women with and without preeclampsia are shown in Table 1. The baseline characteristics including maternal age, proportion of women who were at an advanced maternal age (35 years old or more at the estimated date of confinement), parity, prepregnancy body mass index (BMI), proportion of obese women (defined as prepregnancy BMI 30 kg/m^2^ or more), total weight gain and gestational age at measurement were not significantly different between the two groups. However, preeclamptic women had a significantly higher mean arterial blood pressure at the first trimester than non-preeclamptic women (90.8 ± 9.7 vs. 83.1 ± 8.4 mmHg, p < 0.001). On the other hand, for pregnancy and neonatal outcomes, pregnant women who developed preeclampsia had a lower gestational age at delivery (36.6 ± 2.5 vs. 38.2 ± 1.5 weeks, p < 0.001), higher preterm delivery rate (22.6% vs. 5.6%, p = 0.003), lower neonatal birth weight (2747.3 ± 631.6 vs. 3116 ± 440.8 g, p < 0.001), higher rate of low birth weight (< 2500 g) (22.6% vs. 5.6%, p = 0.001), lower Apgar scores at the 1st and 5th-minute after birth (8.1 ± 1.8 vs. 8.9 ± 0.5, p < 0.001 and 9.3 ± 1.8 vs. 9.9 ± 0.3, p < 0.001, respectively), higher rate of neonatal respiratory distress syndrome (RDS) (19.4% vs. 1.7%, p < 0.001), and longer length of hospital stay (6.7 ± 8.3 vs. 4.2 ± 2.8 days, p < 0.001) than non-preeclamptic women.

**Table 1:** Baseline characteristics and pregnancy outcomes of women with preeclampsia and controls.

	Controls (n = 354)	Preeclampsia (n = 31)	p value
Maternal age (years)	32.8 ± 4.9	33.3 ± 4.6	0.612
Advanced maternal age (≥ 35 years old)	135 (38.1)	15 (48.4)	0.262
Primigravida	173 (48.9)	17 (54.8)	0.523
**Parity**			0.537
0	202 (57.1)	20 (64.5)	
≥ 1	152 (42.9)	11 (35.5)	
Prepregnancy BMI (kg/m^2^)	22.6 ± 4.5	23.6 ± 3.6	0.197
Obesity (BMI ≥ 30 kg/m^2^)	26 (7.3)	0 (0)	0.25
Total weight gain (kg)	13.2 ± 4.7	14.6 ± 4.3	0.106
GA at measurement (weeks)	12.3 ± 0.7	12.4 ± 0.6	0.241
Mean arterial pressure (mmHg)	83.1 ± 8.4	90.8 ± 9.7	< 0.001
Gestational diabetes	22 (6.2)	5 (16.1)	0.088
Fetal growth restriction	2 (0.6)	2 (6.5)	0.034
GA at delivery (weeks)	38.2 ± 1.5	36.6 ± 2.5	< 0.001
Delivery at GA < 37 weeks	20 (5.6)	7 (22.6)	0.003
Delivery at GA < 34 weeks	5 (1.4)	2 (6.5)	0.102
**Mode of delivery**			0.499
Vaginal delivery	154 (43.5)	11 (35.5)	
Cesarean section	200 (56.5)	20 (64.5)	
Birth weight (g)	3116 ± 440.8	2747.3 ± 631.6	< 0.001
Low birth weight (< 2500 g)	20 (5.6)	7 (22.6)	0.001
**Apgar score**			
1 min	8.9 ± 0.5	8.1 ± 1.8	< 0.001
5 min	9.9 ± 0.3	9.3 ± 1.8	< 0.001
Neonatal respiratory distress syndrome	6 (1.7)	6 (19.4)	< 0.001
Perinatal death	1 (0.3)	1 (3.2)	0.155
Length of hospital stay	4.2 ± 2.8	6.7 ± 8.3	< 0.001

Data are presented as mean ± SD or n (%).

*BMI* body mass index, *GA* gestational age.

The median (IQR) serum HIF-1α levels of overall preeclampsia and late-onset preeclampsia were 1315.2 (645.9, 5128.7), and 1715.5 (852, 5134) pg/ml, respectively, which were significantly higher than that of normal pregnant women (699.5 (426.8, 1107.1) pg/ml) (p < 0.001, and < 0.001, respectively). However, the median (IQR) serum HIF-1α level of early-onset preeclampsia was 646.7 (438.9, 5151.9) pg/ml which was not different compared to the controls (p = 0.629). There was no differences in the mean PI of the uterine artery Doppler between women with preeclampsia and control women (p = 0.775). However, the mean PI of the uterine artery Doppler was significantly higher in women with early-onset preeclampsia than healthy control women (2.21 ± 0.47 vs. 1.72 ± 0.48, p = 0.013). The mean PI of the uterine artery Doppler was comparable between late-onset preeclampsia women and the healthy control women (Table 2).

**Table 2:** Serum HIF-1α levels and UtA Doppler PI in women with preeclampsia, early-onset preeclampsia, and late-onset preeclampsia compared to healthy controls.

	Controls (n = 354)	Preeclampsia (n = 31)	Early-onset preeclampsia (n = 6)	Late-onset preeclampsia (n = 25)	p value
HIF-1α (pg/ml)	699.5 (426.8, 1107.1)	1315.2 (645.9, 5128.7)			< 0.001
			646.7 (438.9, 5151.9)		0.629
				1715.5 (852, 5134)	< 0.001
UtA PI	1.72 ± 0.48	1.74 ± 0.48			0.775
			2.21 ± 0.47		0.013
				1.63 ± 0.41	0.38

Data are presented as median (IQR), mean ± SD or n (%).

*HIF-1α* hypoxia-inducible factor-1 alpha, *UtA PI* uterine artery pulsatility index.

**Figure 1:**
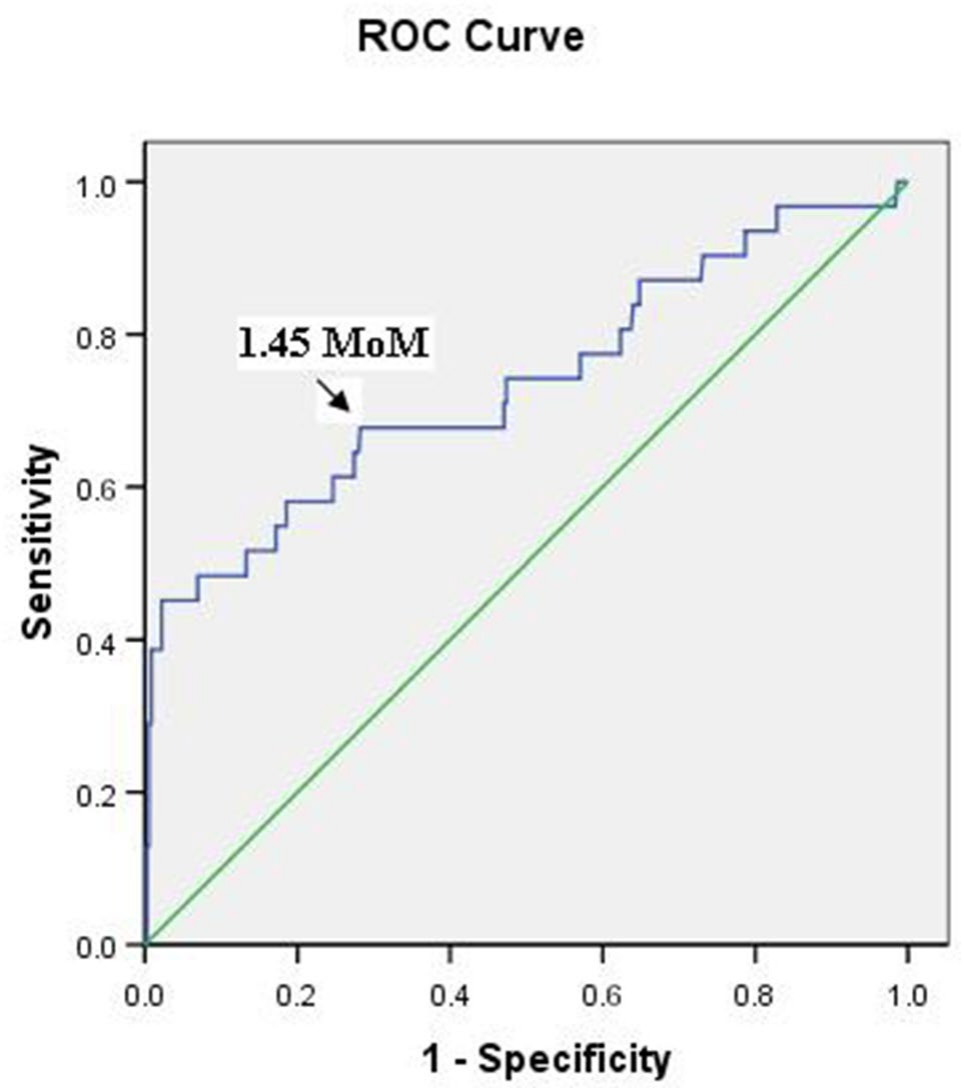
Receiver-operating characteristic curve for the relationship between the serum hypoxia-inducible factor-1α level and the diagnosis of preeclampsia (area under the curve: 0.735; 95% confidence interval: 0.625–0.844; p < 0.001).

The optimal cut-off value of the serum HIF-1α levels, calculated from the receiver operating characteristic curve (AUC = 0.735, p < 0.001), was 1.45 multiple of median (MoM), according to the gestational age at the time of measurement (Fig. 1). The sensitivity, specificity, PPV, and NPV were 66.7%, 71.5%, 17.2%, and 96.2%, respectively, when using serum levels above the cut-off value for predicting preeclampsia. To predict early-onset preeclampsia, the sensitivity, specificity, PPV, and NPV were 33.3%, 68.3%, 1.6%, and 98.5%, respectively (Table 3).

**Table 3:** Predictive value of serum HIF-1α levels and UtA Doppler for preeclampsia.

	Sensitivity (%)	Specificity (%)	PPV (%)	NPV (%)	Positive LR	Negative LR
**Overall preeclampsia**						
HIF-1α levels > 1.45 MoM	66.7 (48.6, 83.3)	71.5 (66.5, 76.1)	17.2 (13.4, 21.8)	96.2 (93.8, 97.7)	2.4 (1.8, 3.2)	0.5 (0.3, 0.8)
UtA PI > 95th percentile	6.5 (0.8, 21.4)	95.2 (92.4, 97.2)	10.5 (2.8, 32.7)	92.1 (91.4, 92.7)	1.3 (0.3, 5.6)	0.9 (0.8, 1.1)
Abnormal HIF-1α levels and/or UtA PI	74.2 (55.4, 88.1)	67.2 (62.1, 72.1)	16.6 (13.3, 20.4)	96.8 (94.2, 98.2)	2.3 (1.8, 2.9)	0.4 (0.2, 0.7)
**Early-onset preeclampsia**						
HIF-1α levels > 1.45 MoM	33.3 (4.3, 77.7)	68.3 (63.4, 72.9)	1.6 (0.5, 4.9)	98.5 (97.3, 99.1)	1.1 (0.3, 3.3)	0.9 (0.6, 1.7)
UtA PI > 95th percentile	16.7 (0.4, 64.1)	95.3 (92.6, 97.2)	5.3 (0.9, 26)	98.6 (98.1, 99)	3.5 (0.6, 22.2)	0.9 (0.6, 1.3)
Abnormal HIF-1α levels and/or UtA PI	50 (11.8, 88.2)	64.1 (59.1, 68.9)	2.2 (1, 4.7)	98.8 (97.3, 99.5)	1.4 (0.6, 3.1)	0.8 (0.4, 1.7)
**Late-onset preeclampsia**						
HIF-1α levels > 1.45 MoM	76 (54.9, 90.6)	71.4 (66.4, 76)	15.6 (12.3, 19.5)	97.7 (95.5, 98.9)	2.7 (2, 3.5)	0.3 (0.2, 0.7)
UtA PI > 95th percentile	4 (0.1, 20.4)	95 (92.2, 97)	5.3 (0.8, 28.5)	93.4 (92.9, 93.9)	0.8 (0.1, 5.8)	1 (0.9, 1.1)
Abnormal HIF-1α levels and/or UtA PI	80 (59.3, 93.2)	66.9 (61.8, 71.8)	14.4 (11.6, 17.7)	97.9 (95.6, 99.1)	2.4 (1.9, 3.1)	0.3 (0.1, 0.7)

*HIF-1α* hypoxia-inducible factor-1 alpha, *UtA PI* uterine artery pulsatility index, *PPV* positive predictive value, *NPV* negative predictive value, *LR* likelihood ratio, *MoM* multiple of median.

The 95th percentile of the mean uterine artery Doppler PI in this study, stratified into three groups according to the gestational age at the time of measurement, were 2.77, 2.72, and 2.38 at 11–11^+6^, 12–12^+6^, and 13–13^+6^ weeks, respectively. Using the mean uterine artery Doppler PI above the 95th percentile as a cut-off value for predicting preeclampsia, the sensitivity, specificity, PPV, and NPV were 6.5%, 95.2%, 10.5%, and 92.1%, respectively. To predict early-onset preeclampsia, the sensitivity, specificity, PPV, and NPV were 16.7%, 95.3%, 5.3%, and 98.6%, respectively (Table 3).

A combination of abnormal serum HIF-1α levels (higher than 1.45 MoM) and abnormal uterine artery Doppler PI (above the 95th percentile) was used as a predictive value for preeclampsia and its sensitivity, specificity, PPV, and NPV were 74.2%, 67.2%, 16.6%, and 96.8%, respectively. A combination of abnormal serum HIF-1α levels (higher than 1.45 MoM) and abnormal uterine artery Doppler PI (above the 95th percentile) was used as a predictive value for early-onset preeclampsia and its sensitivity, specificity, PPV, and NPV were 50%, 64.1%, 2.2%, and 98.8%, respectively (Table 3).

The relative risk of other pregnancy complications in the participants with an abnormal serum HIF-1α levels and/or abnormal uterine artery PI compared to the controls is shown in Table 4. Preterm delivery and neonatal respiratory distress syndrome were significantly different between preeclamptic women and non-preeclamptic women.

**Table 4:** Predictive value of serum HIF-1α levels and UtA Doppler for other pregnancy complications.

	Relative risk	95% Confidence interval
Fetal growth restriction	1.848	0.693–4.925
Preterm delivery	1.259	1.006–1.577
Gestational diabetes	1.138	0.949–1.366
Neonatal respiratory distress syndrome	1.866	1.059–3.288
Perinatal death	1.843	0.461–7.373

*HIF-1α* hypoxia-inducible factor-1 alpha, *UtA* uterine artery.

## Discussion

This study found that the serum HIF-1α levels with or without the uterine artery Doppler in the first trimester was effective in predicting preeclampsia. The sensitivity of serum HIF-1α levels with or without the uterine artery Doppler in predicting preeclampsia were 74.2% and 66.7%, respectively.

In the present study, the serum HIF-1α levels were significantly higher in preeclamptic women compared to non-preeclamptic women. Previous studies showed that women with preeclampsia had persistent elevated placental HIF-1α levels that enhanced the transcription of genes that encoded soluble fms-like tyrosine kinase-1 (sFlt-1), soluble endoglin (sEng), and endothelin-1 (ET-1), all known to contribute to preeclampsia^11,16–18^. Rath et al.’s study^19^ found significant upregulated concentration of HIF-1α not only in the placental tissues but also in the serum samples of preeclamptic woman collected at the time of diagnosis (mean 6.581 pg/ml in preeclamptic women vs. 4.947 pg/ml in healthy control women, p = 0.0001). However, the discrepancy of the mean serum HIF-1α levels between this study and that observed in Rath et al.^19^ might be due to the difference in the study population and gestational age at measurement.

In this study, serum HIF-1α concentrations were higher in late-onset preeclamptic women than early-onset preeclamptic ones. HIF-1α may be a marker of late-onset preeclampsia as HIF-1α has been described as a marker of cardiovascular disease^24^.

The incidence of overall and early-onset preeclampsia in this study was higher than expected. This may be due to the high number of advanced maternal age in the study. Advanced maternal age is one of the risk factor for preeclampsia^25^.

The mean PI of the uterine artery Doppler was significantly higher in women with early-onset preeclampsia. Neither overall preeclampsia nor late-onset preeclampsia showed any significant differences in mean PI of the uterine artery Doppler when compared to the controls. This observation was similar to previous studies^26,27^. In this study, the use of uterine artery Doppler to predict preeclampsia and early-onset preeclampsia appeared to have high specificity (95.2% and 95.3%, respectively) but low sensitivity (6.5% and 16.7%, respectively). Similarly, Velauthar et al.’s meta-analysis^28^ as well as Townsend et al.’s review^10^ concluded that the first-trimester uterine artery Doppler may be used as a predictor for preeclampsia because of its high specificity (92.1% and 93.4%, respectively) even though the sensitivity was low (47.8% and 26.4%, respectively).

The results of this study revealed that the serum HIF-1α level had a good predictive value when used alone or in combination with uterine artery Doppler to predict preeclampsia during the first trimester screening process. The result was similar to a previous study that found a combination of Doppler of uterine artery with maternal serum markers had good sensitivity for predicting preeclampsia during the first trimester^26^. On the other hand, previous studies found that a combination of Doppler of uterine artery with maternal serum markers had poor sensitivity and specificity in predicting preeclampsia during the first trimester. But it could only predict early-onset preeclampsia^27,29^. This discrepancy may be due to the different serum markers used to detect preeclampsia. In addition, the sensitivity to predict early-onset preeclampsia in this study was not good when compared to previous studies^27,29^. Once again, this may be due to the different serum markers used. It is also possible that the pathogenesis between early and late-onset preeclampsia are different. Some biomarkers are good in detecting certain type of preeclampsia.

Regarding first trimester angiogenic factors and HIF-1α in preeclampsia, there has been no study to evaluate first trimester angiogenic factors as their expression is enhanced by increased HIF-1α. Further studies should be conducted to evaluate first trimester angiogenic factors and HIF-1α in preeclampsia.

The strength of this study was that this is the first prospective study that used serum HIF-1α levels combined with uterine artery Doppler in the first trimester to predict preeclampsia. Early screening of preeclampsia by using a combination of serum HIF-1α levels and uterine artery Doppler during the first trimester (11–13^+6^ weeks of gestation) allows the timing for using early low-dose aspirin prophylaxis in order to prevent preeclampsia, which may be more effective if started prior to 16 weeks of gestation^7^. Moreover, these two screening tests can be performed at the same visit and at the time of the first trimester ultrasonographic screening which will be convenient for the patients. The limitation of this study was that there were small cases of early-onset preeclampsia. Additional studies with a larger sample size of early-onset preeclampsia and other models using serum HIF-1α levels combined with uterine artery Doppler, maternal characteristic risk factors, or other biomarkers should be conducted.

## Conclusion

The serum HIF-1α levels with or without the uterine artery Doppler at 11–13^+6^ weeks of gestation were effective in predicting preeclampsia.
